# Biomechanical Analysis of Concealed Pack Load Influences on Terrorist Gait Signatures Derived from Gröbner Basis Theory

**Published:** 2014-10-14

**Authors:** Sean S Kohles, Anum Barki, Kimberly D. Kendricks, Ronald F. Tuttle

**Affiliations:** 1 Division of Biomaterials & Biomechanics Department of Restorative Dentistry Oregon Health & Science University Portland.; 2 Department of Engineering Physics Air Force Institute of Technology, Wright Patterson Air Force Base.; 3 Department of Mathematics and Computer Science Center for Human Performance and Sensor Applications Central State University Wilberforce.; 4 Department of Engineering Physics Air Force Institute of Technology, Wright Patterson Air Force Base.

**Keywords:** Biomechanics, Kinesiology, Counter-terrorism, Gait parameters, Concealed pack loads, Inverse dynamics

## Abstract

This project examines kinematic gait parameters as forensic predictors of the influence associated with individuals carrying concealed weighted packs up to 20% of their body weight. An initial inverse dynamics approach combined with computational algebra provided lower limb joint angles during the stance phase of gait as measured from 12 human subjects during normal walking. The following paper describes the additional biomechanical analysis of the joint angle data to produce kinetic and kinematic parameters further characterizing human motion. Results include the rotational velocities and accelerations of the hip, knee, and ankle as well as inertial moments and kinetic energies produced at these joints. The reported findings indicate a non-statistically significant influence of concealed pack load, body mass index, and gender on joint kinetics (p>0.05). Ratios of loaded to unloaded kinematics, however, identified some statistical influence on gait (p<0.05). On-going studies are examining an additional subject cohort with greater pack loads in an effort to identify alterations in gait signatures as a counter-terrorism approach.

## Introduction

Suicide bombings have become an increasing threat to civilians and military personnel in Middle Eastern countries. In a recent study that documented civilian casualties from suicide attacks during the Iraq War from 2003 to 2010, over thirty thousand civilian injuries and over twelve thousand civilian deaths were caused by such bomb events [1]. The article also reported deaths of 200 coalition troops of which 175 were members of the United States military. An online database has also documented the reported deaths of Iraqi civilians from the first day of the Iraq War [2]. Overall, as of December 17, 2012 an average of 7.5 civilians have died each day due to ‘terror attacks’ in Iraq, demonstrating threat to civilian and military personnel. To address this threat, biometrics such as fingerprint, face, iris, and gait data have been used successfully to identify suspicious individuals [3–6]. Of these, gait is the least intrusive, and can be studied using video surveillance at a distance. However, gait characteristics are more difficult to identify and isolate when compared to fingerprints or facial features [7]. As a response to these challenges, the investigation into human gait characteristics for the purpose of developing an automatic human identification system was initiated through the INSPIRE project (Integration of a Sensor Package for Identifying Radical Extremists). One of the objectives within INSPIRE is to design a suicide bomber vetting system that is able to screen individuals 150 to 300m away from a military or civilian checkpoint through the use of video captured data. The goal in the present work is to measure and identify human gait characteristics within a controlled laboratory setting that may be used as a possible ‘gait signature’ for an individual carrying a concealed detonation device having recognizable weight.

The human eye is sensitive to motion stimuli and can detect the gait characteristics of familiar individuals such as family and friends, particularly in crowds [8,9]. Yet, it is not clear what gait markers the human visual system is using for individual identification. An aim of this issue is to isolate motion perception to determine these gait markers. Research studies using point-light displays of human motion have demonstrated improvements in identifying observed motion characteristics, and the reliability of these observations has increased [10–12].

This study addresses this issue by examining the data collected from point-light displays of the lower extremities when additional load is carried on the body to identify altered markers in the gait cycle. This effort further examines the mechanics of extension and flexion of the lower extremity joints during human motion beyond that previously reported [13]. The anatomic functions that have been analyzed include extension and flexion at the hip and knee joint well as plantar and dorsi flexion at the ankle joint. A three-link, segmental model has provided a simplified arrangement to examine human dynamic walking, a biped gait pattern which is considered dynamically stable [14]. The application of a rigid-body dynamics analysis has been shown to effectively facilitate the description of human motion, which is periodic, stable, and energy efficient. The human body represents a well-balanced walking machine with highly sophisticated mechanics and control. The working hypothesis in this investigation is that the controlled motion of gait can be perturbed when individuals wear concealed and weighted packs, when compared to a no-load condition. In reality, most terrorist subjects would likely not be identified during the no-load condition. The objective here is to validate the sensitivity in this analytical approach by defining the key mechanics of lower limb motion, which may be used as markers indicating any gait perturbations caused by an increase in concealed pack loads.

## Materials and Methods

### Data Collection

**Figure 1:**
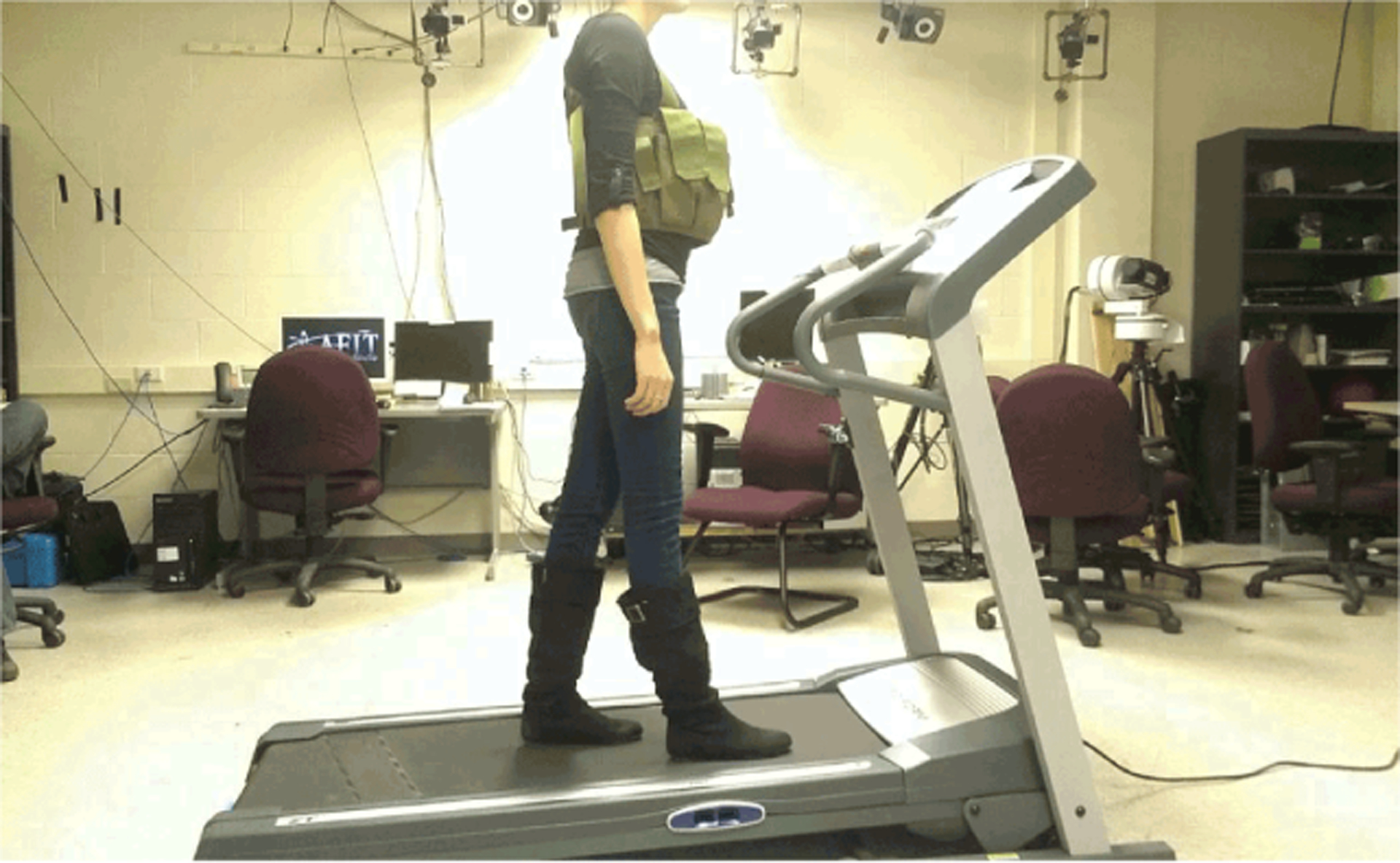
Representative image of a subject wearing a loaded ammunition pack while walking on the laboratory treadmill. There was no slope to the walking surface for this particular study (0° incline setting). Note that the pack load is distributed about the subject’s mid-section with front-load pockets, near their body center of mass (cm _Body_).

Lower limb anatomic joint location data were collected from 12 healthy subjects between the ages of 21 and 42 (7 males and 5 females) using a three-dimensional motion capture system (MX T160 cameras and Nexus software, Vicon Motion Systems Ltd.). Subjects were instructed to ambulate at a comfortable, self-selected walking speed on a treadmill with zero incline (Figure 1), and based on a previous gait study [15]. Joint location data were tracked unilaterally through time at 30 Hz (Δt = 0.03 s/frame) with reflective markers attached to skin-tight outer garments [16]. Each marker was carefully attached on the skin near a palpable bony prominence associated with the four lower limb joints of interest. These locations helped to reduce the noise that may be created by clothing or soft tissue artifacts [17,18]. Marker 1 was placed on the hip joint near the femoral greater trochanter, marker 2 was placed at the lateral fibular condyle near the knee joint, marker 3 was placed at the lateral malleolus near the talocrural ankle joint, and marker 4 was placed laterally near the metatarsalphalangeal joint at the head of the fifth metatarsal.

The subjects were instructed to walk for ten gait cycles leading up to a single measurement cycle (average walking speed range = 2.1 to 3.6 mi/h or 0.94 to 1.61 m/s). Each subject wore a six-pocket vest representing a concealable pack design allowing for multiple weight distributions. The pack design and pack loads followed that of previous study examining sagittal plane stability during gait [19]. After the initial no-load trial, a 10% body weight (BW) load was added to the vest (Figure 1). Trials continued with each trial adding 5% BW up to 20% BW (maximum carried = 26 lbs or 11.8 kg mass) as approved by the AFIT Institutional Review Board (IRB). Each subject experienced 2 to 4 total gait trials. Gait data were analysed from one representative stance phase during the gait cycle. The body mass index (BMI) was determined for each subject as a generalized metric of physical health such that BMI is equal to body mass (in kg) divided by the square of the subject’s height (in m). A normal BMI range for adults is 18.5 to 24.9 [20].

### Joint Angle Determination from Gröbner Basis Theory

**Figure 2:**
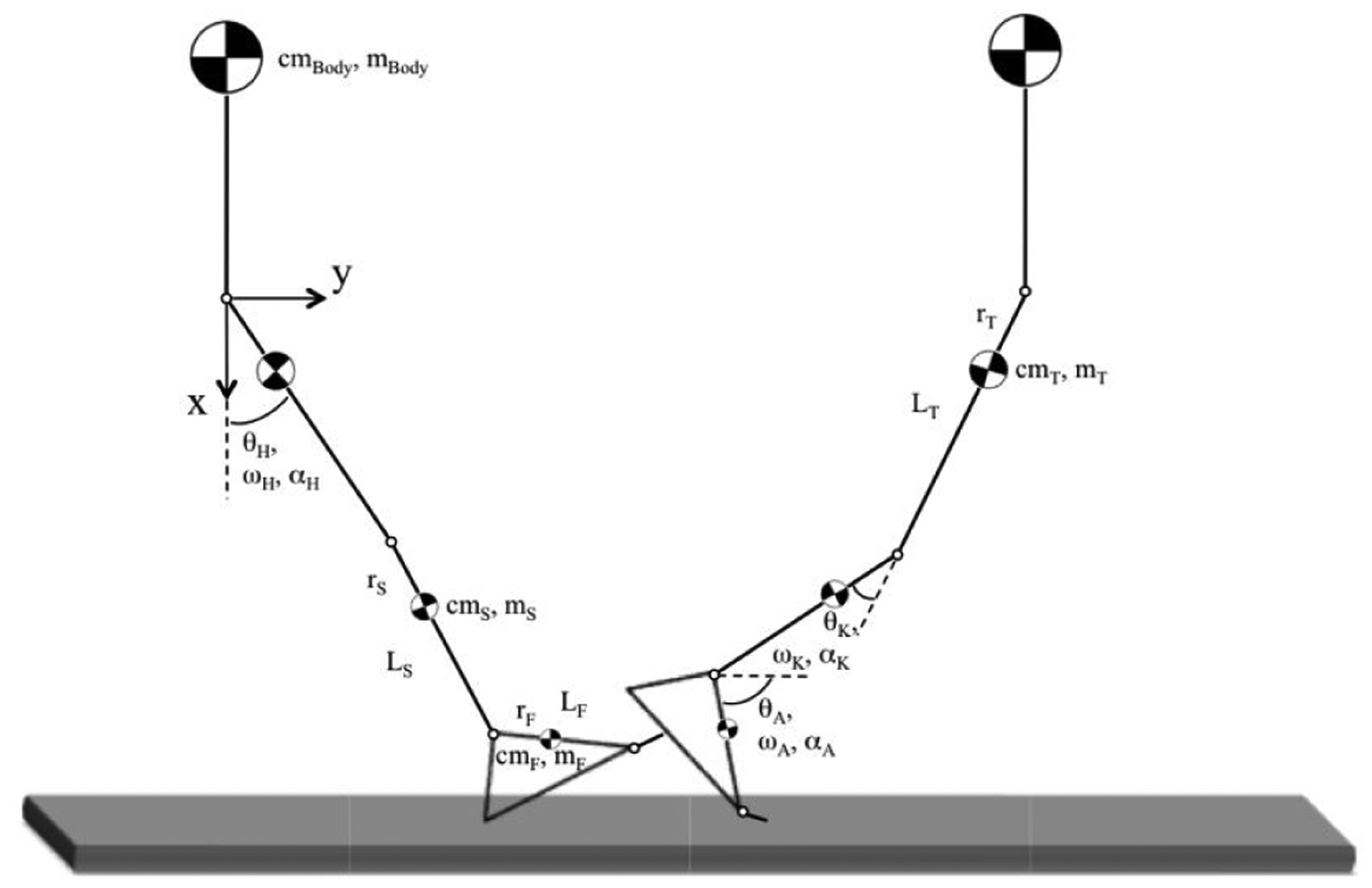
Schematic representing a single-limb, human multisegmental model during the heel-touch (left) to toe-off (right) phase of gait as analyzed in this work. Segmental lengths (L_k_), masses (m_k_), centers of mass (cm_k_), and locations (r_k_) of each cmk are indicated along with the positive angular sign-conventions for displacement (θ_j_) and velocity (ω_j_) drawn relative to each respective joint datum (dashed lines).

A previously developed and validated gait model for lower [13,16,21] and upper [22,23] extremity limb motion was applied to the gathered joint position data. This model facilitated the analysis of joint angles through known limb segment lengths gathered from each subject (Figure 2). The inverse dynamics approach was applied through engaging Gröbner Basis Theory to a single stance-phase of the gait cycle (heel-touch to toe-off). Briefly, the Gröbner basis computation was introduced in 1965 as an algebraic tool for solving a set of non-zero polynomial equations [24]. The computation is a generalization of both Euclid’s algorithm and Gaussian elimination [25]. The computational algorithm was applied here to a set of multivariate nonlinear equations having in common certain properties such as the linked anatomic geometries and motions captured during gait. This approach allowed for a simplified algebraic solution to the inverse kinematics problem as a preface to further biomechanical analyses described herein.

### Joint Kinetic and Kinematic Analysi0073

Euler rotational velocities (ω) and accelerations (α) about the horizontal z-axes (r-θ polar coordinates defining the sagittal plane) through each of the lower limb joints were determined [26]. These kinematic parameters were calculated from the joint angular displacements (Δθ in radians) and time data as determined from a previous Gröbner basis computation [16]. The calculations of angular velocity and accelerations were based on 2Δt instead of Δt, so that the angular velocity (ω_i_) and the angular acceleration (α_i_) at each i^th^ sample were:

(1) $$\omega_{\text{i}}=\left(\theta_{\text{i}+1}-\theta_{\text{i}-1}\right)/2\;\Delta \text{t and }\alpha_{\text{i}}=\left(\omega_{\text{yi}+1}-\omega_{\text{yi}-1}\right)/2\Delta \text{t}$$

where Δt is the time between sequential motion capture frames and defined above as 0.03s.

The net reaction moments (joint, weight, and ground reaction) can be calculated by applying the rotational analogue of Newton’s Second Law of Motion (∑M = Iα). In this study, no ground reaction forces were gathered, thus the isolated joint moments themselves cannot be determined. However, the inertial moments for each i^th^ time sample about each j^th^ joint (j = hip, knee, ankle) can be defined for each k^th^ limb segment (k = thigh, shank, foot) as:

(2) $$\sum {\text{M}}_{\text{j}}={\text{I}}_{\text{k}}\alpha_{\text{i}}$$

Based on percentile limb segment masses (m_k_) from which limb segment mass moments of inertia (I) can be calculated about each joint [27,28]. From the anthropometric data, the following values were calculated from published limb mass distributions [28] and related to polar reference axes with origins at either the hip, knee, or ankle joints (Figure 2): Mass of the thigh (m_T_); Mass of the shank (m_S_); Mass of the foot (m_F_); Lower limb total mass (m_LL_ = m_T_ + m_S_+ m_F_); Center of mass from the proximal limb end of the thigh (cm_T_); Center of mass from the proximal limb end of the shank (cm_S_); Center of mass from the proximal limb end of the foot (cm_F_); and the polar coordinate distances of the cm_T_, cm_S_, and cm_F_ from the joint origins (r_T_, r_S_, and r_F_, respectively). The limb segment lengths were measured from each subject directly (L_T_, L_S_, and L_F_).

In the calculation of the mass moment of inertia for the lower limb segments, the non-uniformity of the limb and each segment was accounted for via the center of mass placements:

(3) $${\text{Icm}}_{\text{k}}=\int {\text{r}}^{2}\text{dm}=\int {\text{r}}^{2}\left({\text{m}}_{\text{k}}/{\text{L}}_{\text{k}}\right)\;\text{dr}$$

The parallel axis theorem then allows for the moment of inertia to be calculated about any joint axis, where D_k_ is the distance from the joint center of rotation to the cm_k_:

(4) $$\text{I}=\sum {\text{Icm}}_{\text{ k}}+\sum {\text{m}}_{\text{ k }}{{\text{D}}^{2}}_{\text{ k}}$$

Here, the summation indicates that each distal segment will contribute to the inertia about the proximal joint, i.e., the hip joint mechanics are influenced by the inertia of the thigh, knee, and foot, etc.

Upon determination of the limb segment inertia, rotational kinetic energies (KE), which can be derived from the integration of the inertial moment in equation (2), were also determined for each joint at each time increment throughout the entire stance phase:

(5) $${\text{KE}}_{\text{j}}=½{\text{I}}_{\text{j}}{\omega^{2}}_{\text{i}}$$

Thus, directly incorporating the rotational velocity kinematics.

### Statistical Analysis

Extrema kinematic parameters were identified during functional joint motion and used to calculate the respective maxima (largest positive) and minima (largest negative) kinetic parameters. Descriptive and comparative statistics including analysis of variance (one-way, two-way and three-way ANOVAs) tested the statistical influence of the primary objective of pack weight on the kinetic and kinematic gait parameters during the repeated measures design. Missing data within the experimental design were accounted for within the ANOVAs. Kinematic parameters were normalized for each subject by their non-load trials and analysed in aggregate with a student’s t-test (any-load ÷ no-load distributions compared with a hypothesized mean=1.0). The statistical analysis was also applied to identify the strength of other independent variables (gender, BMI, and anatomic joint) as predictors of the dependent variables (joint angular position, angular velocities, rotational accelerations, inertial moments, and kinetic energy). All data/statistical analyses were conducted with commercial software (JMP v5.0.1, SAS Institute, Inc.). Statistical significance was considered when p<0.05, with α = 0.05 minimizing Type I errors.

## Results

All 12 subjects (mean BMI = 23.33 +/−3.25 sd) contributed to 36 total gait trials. Kinematic and kinetic parameters were determined for each anatomical joint at each time increment throughout the single stance phase of gait. Means +/− standard deviations {sd} are presented in the tables and figures.

### Joint Kinematics

The results from all combined subjects indicate a subtle influence of pack weight on the kinematic metrics of joint position, velocity, and acceleration compared with the no- load condition (Table 1). The ratios represent normalization with the no-load condition for each joint at each load condition. The stated p-values indicate statistical comparison with a hypothesized distribution mean of 1.0 as aggregated over all of the pack loads. A number of the maxima (left column) and minima (right column) kinematic results indicate a statistically significant difference from the no-load equivalence of 1.0 (p<0.05). However, multi-way ANOVAs consistently indicated no statistical influence of either pack load or BMI on gait kinematics (p>0.05).

### Joint Kinetics

**Figure 3:**
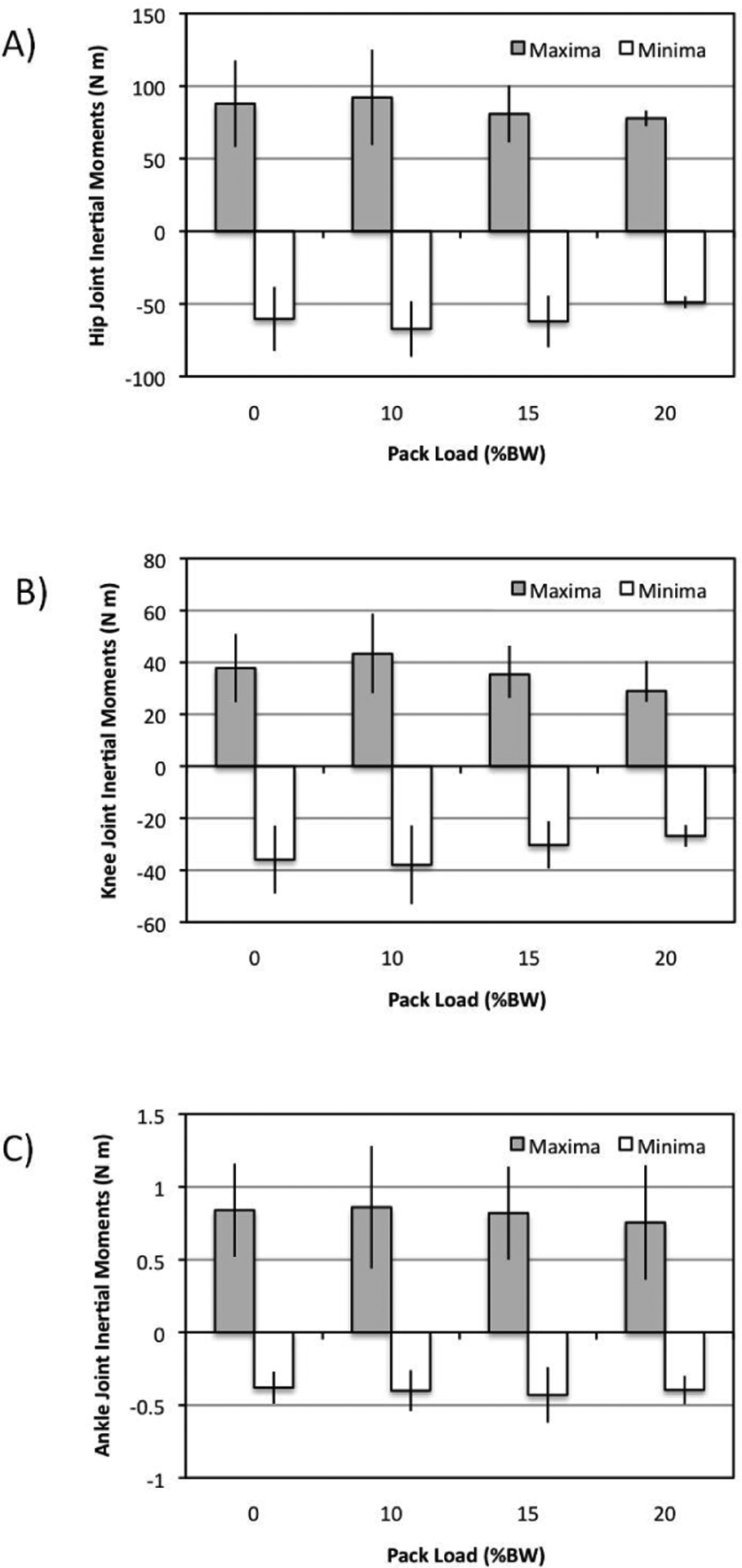
Mean inertial moments {+/− sd} plotted for all subjects over the range of pack loads as determined for the (A) hip, (B) knee, and (C) ankle joints.

**Figure 4:**
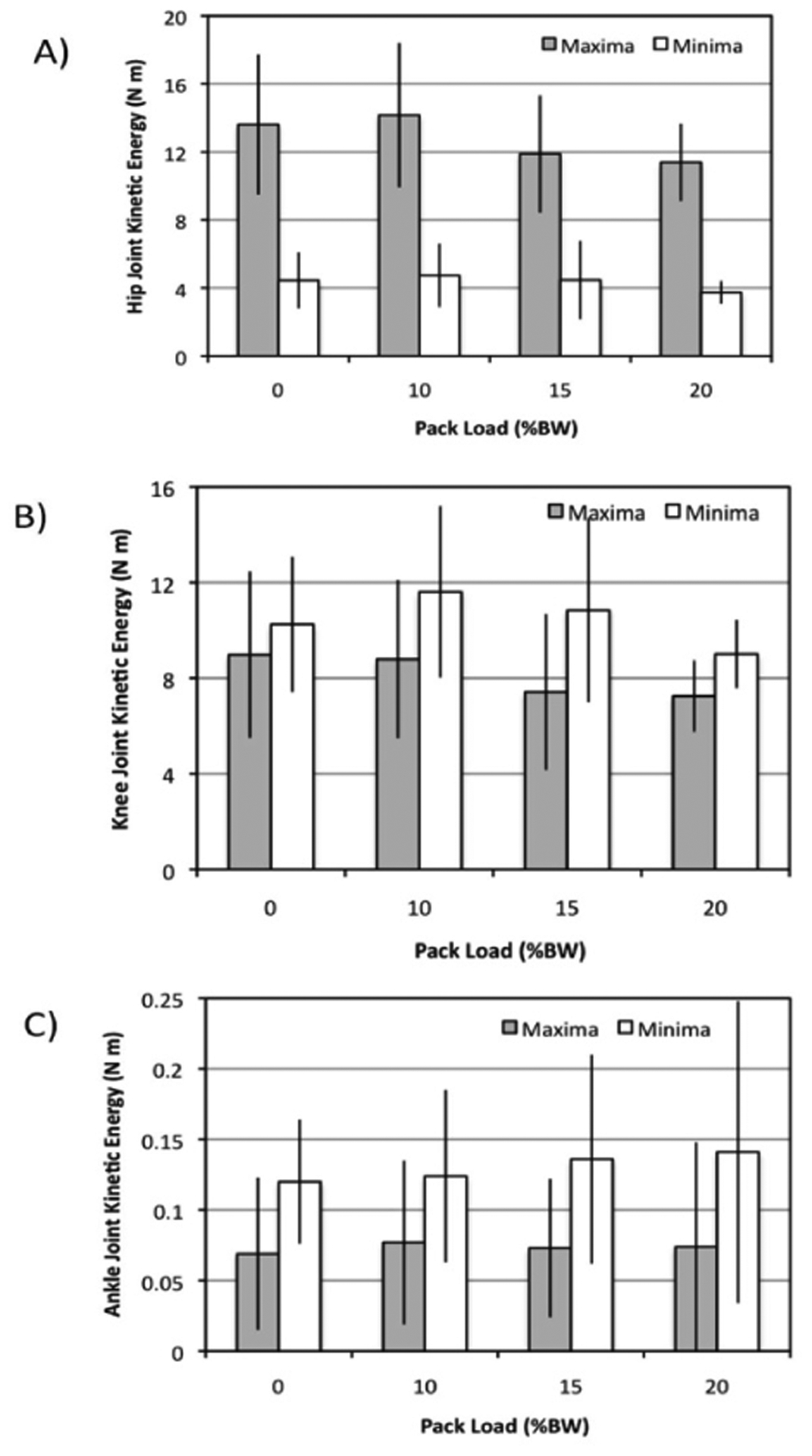
Mean kinetic energies {+/− sd} plotted for all subjects over the range of pack loads as determined for the (A) hip, (B) knee, and (C) ankle joints. Note that kinetic energy is determined from the square of the peak angular velocities.

Findings from all combined subjects indicated little influence (p>0.05) of pack weight on either the inertial moments (Figure 3) or the kinetic energies (Figure 4).

These values were further tabulated separately for male (Table 2) and female (Table 3) subjects as means to explore any gender-dependent characteristics although no statistical difference in kinetics were shown (p>0.05). Again, the split cells within the tables indicate the maxima (left) and minima (right) values generated by the subject during the analysed gait cycle. A three-way ANOVA did however indicate a statistical influence of BMI on joint kinetics, especially that of the inertial moments (Table 4).

## Discussion

The presented work provides a unique perspective on forensic gait biomechanics by investigating the influence of hidden pack weights on human motion. In addition, the study builds off of the novel application of the Gröbner Basis Theory as a streamlined approach to calculating the inverse dynamics associated with human limb and joint motion. The quantified approach demonstrates a potentially useful transition from joint displacement data to full kinematic and kinetic metrics, which may provide further gait characterization as signature motions identifying terrorist activities.

Although the presented results did not provide definitive biomechanical signatures of perturbed gait, the calculated values were similar to the lower limb joint kinematics and kinetics reported by other researchers during studies of walking. As ground reaction forces were neither measured nor incorporated here, inertial-based moments and energy provide only a partial comparison of the joint mechanics [29]. Previous reports indicated that normal walking speeds generated similar motion within the hip, knee, and ankle [30]. As example, mean peak knee flexion was similar (Δ% = 1.4%) between our study (65.9°) and that published previously (65.0°) [30]. In addition, walking speedbased energetics was not as comparable, but within orders of magnitude [31]. Mean peak positive inertial power normalized by body mass and determined at peak inertial moments at the coincident rotational velocities for the hip (1.44 W/kg), knee (2.19 W/kg), and ankle (0.03 W/kg) here can be compared with those powers published for the hip (0.4 W/kg), knee (0.2 W/kg), and ankle (0.5 W/kg), respectively, at similar walking speeds (1.25 m/s) [31]. However, those values incorporated ground reaction forces during an efficiency study. Primarily, what were not shown in the present study were the altered gait parameters associated with increased load carriage as previously reported in the literature using traditional backpack designs. We reported an inconsistent influence of pack load on gait kinetics and kinematics. However, other investigators have shown that subjects walk with greater knee flexion [32–35] and longer stance-phase times [35–38]. These changes were likely due to the much larger loads carried with traditional packs as compared with the pack design and load levels in the present study (range of maximum load differences between this study, (Δ% = 130 to 324%). In addition, we did not measure stance-phase timing or length. The results reported here however have provided a baseline comparison to further clarify this known influence.

The primary strength of this study is in the generation of kinetic parameters through an inverse dynamics approach albeit without the use of force-measuring devices (load cells, force plate, etc.). The obvious limitations in the design of this study include the minimal range in the worn pack loads, pack design, small subject population, and minimal subject participation, i.e., only one male subject wore a pack of 20% BW. In response to this limitation, a future study has been approved by our IRB and is underway to incorporate up to 50 subjects each wearing much larger weighted packs. An additional limitation here includes the focus on only two-dimensional motion. Although treadmill restricted gait is generally uni-directional, there are still out-of-plane motions that exist including pelvic swing and abducted/adducted motions. Compensation for these z-direction motions were made during the initial construction of the Gröbner bases by incorporating limb shortening as characterized during motion capture. The two-dimensional approach provided an initial validation of the analytical technique.

This work represents an initial effort in testing the overall objective of whether the measured and calculated biomechanical gait parameters are indeed sensitive to pack load perturbations. A future study has been approved for increasing the upper limits of the pack loads. The analytical protocol described here will be applied toward that gathered data in a continuing effort to forensically identify gait signatures. The implications of these and future findings will be used to minimize the on-going impact of suicide bombings by identifying individuals wearing such incendiary devices.

## Figures and Tables

**Table 1:** Mean kinematic descriptions of motion {+/− sd} normalized by the unloaded pack condition for all study subjects. P-values indicate the t-test comparison with a hypothesized distribution mean of 1.0 aggregated over all three pack loads where statistically significant differences are noted when p < 0.05.

Kinematic Parameter	Load (%BW)	Hip Joint Ratios		Knee Joint Ratios		Ankle Joint Ratios	
		Max	Min	Max	Min	Max	Min
Angular Position (θ)		p = 0.0053	p = 0.2790	p = 0.0355	p = 0.0022	p = 0.1819	p = 0.5718
	10%	1.05 {0.06}	1.01 {0.11}	1.03 {0.04}	1.40 {0.69}	4.67 {12.54}	0.94 {0.10}
	15%	1.02 {0.07}	1.02 {0.15}	1.01 {0.06}	1.46 {0.60}	1.52 {1.27}	1.01 {0.21}
	20%	1.05 {0.04}	1.14 {0.16}	1.02 {0.06}	1.40 {0.36}	4.34 {7.35}	1.04 {0.18}
Angular Velocity (ω)		p = 0.8907	p = 0.1180	p = 0.7113	p = 0.0001	p = 0.0153	p = 0.3664
	10%	1.02 {0.06}	1.03 {0.10}	0.99 {0.06}	1.06 {0.05}	1.05 {0.20}	1.00 {0.13}
	15%	0.97 {0.09}	1.02 {0.13}	0.97 {0.15}	1.04 {0.07}	1.20 {0.31}	1.05 {0.15}
	20%	0.98 {0.07}	1.11 {0.11}	1.05 {0.12}	1.11 {0.03}	1.20 {0.16}	1.06 {0.08}
Angular Acceleration (α)		p = 0.0160	p = 0.0001	p = 0.0179	p = 0.1739	p = 0.0722	p = 0.0354
	10%	1.05 {0.11}	1.14 {0.14}	1.16 {0.24}	1.05 {0.07}	1.00 {0.17}	1.06 {0.22}
	15%	1.05 {0.18}	1.14 {0.17}	1.09 {0.25}	0.99 {0.13}	1.08 {0.14}	1.13 {0.22}
	20%	1.32 {0.15}	1.18 {0.08}	1.12 {0.34}	1.04 {0.06}	1.17 {0.12}	1.16 {0.29}

**Table 2:** Mean kinetic joint parameters {+/− sd} calculated from male subject data (n=7) of which each were carrying a range of pack loads as percentages of body weight. No variance was calculated for the 20% pack load level as data from only one subject were gathered.

Kinetic Parameters	Load (%BW)	Hip Joint		Knee Joint		Ankle Joint	
		Max	Min	Max	Min	Max	Min
Inertial Moment (N m)	0%	99.32 {32.36}	−63.79 {24.65}	44.43 {12.19}	−41.05 {14.28}	0.88 {0.28}	−0.39 {0.11}
	10%	102.26 {38.63}	−72.51 {21.64}	50.14 {11.23}	−43.06 {17.64}	0.94 {0.48}	−0.44 {0.15}
	15%	92.49 {16.86}	−65.96 {23.70}	35.86 {11.48}	−34.25 {11.58}	0.80 {0.22}	−0.50 {0.24}
	20%	83.92	−44.87	41.70	−22.28	0.53	−0.31
Kinetic Energy (N m)	0%	13.45 {4.79}	5.07 {1.72}	9.92 {3.69}	11.29 {3.11}	0.06 {0.03}	0.12 {0.03}
	10%	14.82 {4.95}	5.39 {2.01}	9.76 {3.57}	12.54 {3.86}	0.08 {0.06}	0.13 {0.06}
	15%	12.05 {3.34}	5.38 {3.02}	8.92 {3.70}	12.08 {5.21}	0.05 {0.02}	0.11 {0.05}
	20%	10.67	4.43	6.53	8.55	0.02	0.09

**Table 3:** Statistically similar to male data (p > 0.05), mean kinetic joint parameters {+/− sd} were calculated from female subject data (n=5) of which each were carrying a range of pack loads as percentages of body weight.

Kinetic Parameters	Load (%BW)	Hip Joint		Knee Joint		Ankle Joint	
		Max	Min	Max	Min	Max	Min
Inertial Moment (N m)	0%	71.70 {17.62}	−55.60 {19.36}	28.46 {7.90}	−28.68 {7.14}	0.78 {0.40}	−0.36 {0.12}
	10%	78.10 {17.43}	−60.06 {13.70}	33.73 {16.65}	−30.66 {7.16}	0.74 {0.34}	−0.34 {0.15}
	15%	71.44 {17.45}	−58.98 {13.59}	35.01 {11.91}	−27.03 {5.95}	0.84 {0.41}	−0.37 {0.15}
	20%	74.74 {1.70}	−50.92 {3.09}	22.66 {4.45}	−28.99 {2.18}	0.87 {0.48}	−0.44 {0.09}
Kinetic Energy (N m)	0%	13.85 {3.49}	3.59 {1.17}	7.66 {3.01}	8.79 {1.72}	0.08 {0.08}	0.13 {0.06}
	10%	13.26 {3.32}	3.84 {1.33}	7.44 {2.66}	10.31 {3.09}	0.07 {0.06}	0.12 {0.07}
	15%	11.73 {3.92}	3.76 {1.53}	6.22 {2.62}	9.85 {2.57}	0.09 {0.06}	0.16 {0.08}
	20%	11.75 {3.10}	3.40 {0.44}	7.62 {1.91}	9.24 {1.94}	0.10 {0.08}	0.17 {0.14}

**Table 4:** Statistical results summarizing a three-way ANOVA comparison between the interactions of pack load, anatomical joint, and BMI. While accounting for joint and BMI, there appears to be little influence of the range of pack loads on the kinetic parameters (p > 0.05). However, BMI does appear to be a potential predictor of joint inertial moments (p < 0.05).

	Maximum Kinetic Energy	Minimum Kinetic Energy	Maximum Inertial Moment	Minimum Inertial Moment
Pack Load	p=0.4622	p=0.5145	p=0.6368	p=0.5192
Joint	p=0.0001	p=0.0001	p=0.0001	p=0.0001
BMI	p=0.1130	p=0.7639	p=0.0455	p=0.0220
